# Ascorbate induces apoptosis in melanoma cells by suppressing Clusterin expression

**DOI:** 10.1038/s41598-017-03893-5

**Published:** 2017-06-16

**Authors:** Sushmita Mustafi, David W. Sant, Zhao-Jun Liu, Gaofeng Wang

**Affiliations:** 1 John P. Hussman Institute for Human Genomics, Dr. John T. Macdonald Foundation Department of Human Genetics, University of Miami Miller School of Medicine, Miami, FL 33136 USA; 2 Department of Surgery, University of Miami Miller School of Medicine, Miami, FL 33136 USA; 3 Sylvester Comprehensive Cancer Center, University of Miami Miller School of Medicine, Miami, FL 33136 USA; 4 Dr. Nasser Ibrahim Al-Rashid Orbital Vision Research Center, University of Miami Miller School of Medicine, Miami, FL 33136 USA

## Abstract

Pharmacological levels of ascorbate have long been suggested as a potential treatment of cancer. However, we observed that EC50 of ascorbate was at a similar level for cultured healthy melanocytes and melanoma cells, suggesting a limit of pharmacological ascorbate in treating cancer. Loss of 5-hydroxymethylcytosine (5 hmC) is an epigenetic hallmark of cancer and ascorbate promotes 5 hmC generation by serving as a cofactor for TET methylcytosine dioxygenases. Our previous work demonstrated that ascorbate treatment at physiological level (100 μM) increased 5 hmC content in melanoma cells toward the level of healthy melanocytes. Here we show that 100 µM of ascorbate induced apoptosis in A2058 melanoma cells. RNA-seq analysis revealed that expression of the Clusterin (CLU) gene, which is related to apoptosis, was downregulated by ascorbate. The suppression of CLU was verified at transcript level in different melanoma cell lines, and at protein level in A2058 cells. The anti-apoptotic cytoplasmic CLU was decreased, while the pro-apoptotic nuclear CLU was largely maintained, after ascorbate treatment. These changes in CLU subcellular localization were also associated with Bax and caspases activation, Bcl-xL sequestration, and cytochrome c release. Taken together, this study establishes an impending therapeutic role of physiological ascorbate to potentiate apoptosis in melanoma.

## Introduction

Melanoma is one of the most aggressive forms of cancer that occurs frequently with a significant contribution of environmental factors to its etiology^1^. Aberrant epigenetic alterations, reflected at the interface of a dynamic microenvironment and the genome, are known to be involved in the malignant transformation of melanocytes^2^. Recently, genomic loss of 5-hydroxymethylcytosine (5 hmC) has been found in most, if not all, types of human cancer^3^. 5 hmC is converted from 5-methylcytosine (5 mC), the major epigenetic modification in mammalian DNA, through a process that is catalyzed by Ten-eleven translocation (TET) methylcytosine dioxygenases, which include three members: TET1, TET2 and TET3^4^. TETs can further oxidize 5 hmC to 5-formylcytosine (5 fC) and 5-carboxylcytosine (5caC), which are ultimately replaced by unmodified cytosine to complete cytosine demethylation^5^.

The content of 5 hmC is high in healthy melanocytes but is gradually lost during progression from benign nevi through advancing stages of primary and metastatic melanoma^6–10^. This global loss of 5 hmC disrupts the dynamics of DNA methylation-demethylation and affects genome-wide gene expression, which could eventually lead to malignant transformation. One known mechanism underlying the loss of 5 hmC in some melanoma cases is a decreased expression of TET2 or mutant TET2^6, 11, 12^. Overexpressing TET2 partially re-establishes a normal 5 hmC profile in melanoma cells and decreases their invasiveness^4^. While overexpressing TETs in patients might not be clinically feasible, this discovery suggests that finding a means of restoring normal 5 hmC content may yield a novel therapy for melanoma.

TETs belong to the iron and 2-oxoglutarate (2OG, also known as α-ketoglutarate)-dependent dioxygenase family. They utilize Fe^2+^ as a cofactor and 2OG as a co-substrate. We and others found that ascorbate (ascorbate anion, the dominant form of vitamin C / L-ascorbic acid under physiological pH) acts as a cofactor for TETs to enhance the enzymatic activity of TETs to convert 5 mC to 5 hmC^13–17^. This finding highlights a new function of ascorbate in modulating the epigenetic control of the genome^18^.

Previously, we showed that in addition to downregulated expression of TET2, the level of sodium dependent vitamin C transporters (SVCTs) were also decreased in melanoma cell lines, especially the lines derived from metastatic stage tumors^19^. This is consistent with the report that ascorbate uptake rate by melanoma cells is only ~50% of the uptake rate by healthy melanocytes^20^, suggesting that a shortage of intracellular ascorbate could also underpin the loss of 5 hmC in metastatic melanoma. The average concentration of ascorbate in the plasma of healthy humans is at ~50 μM range and can reach ~150 μM^21^. Treatment of ascorbate at a physiological level (100 μM) increased the content of 5 hmC in melanoma cell lines derived from different stages toward the level of healthy melanocytes, which was comparable to the effect of overexpressing TET2. Ascorbate treatment decreased the malignancy of metastatic A2058 cells by inhibiting migration and anchorage-independent growth, while exerting no obvious effect on proliferation rate^19^.

In the present work, we investigated the impact of ascorbate to induce apoptosis in melanoma cells. We found that ascorbate at a physiological level (100 μM) significantly induced apoptosis in cultured melanoma cells. This effect appeared to be mediated by inhibiting expression of Clusterin (CLU, OMIM 185430), which activates Bax (OMIM 600040), sequesters Bcl-xL (OMIM 600039) in the mitochondria, and releases cytochrome c, further leading to apoptosis. Our results highlight the importance of ascorbate as a potential prevention and treatment for melanoma.

## Results

### Ascorbate Induces Apoptosis in A2058 Melanoma Cells

**Figure 1:**
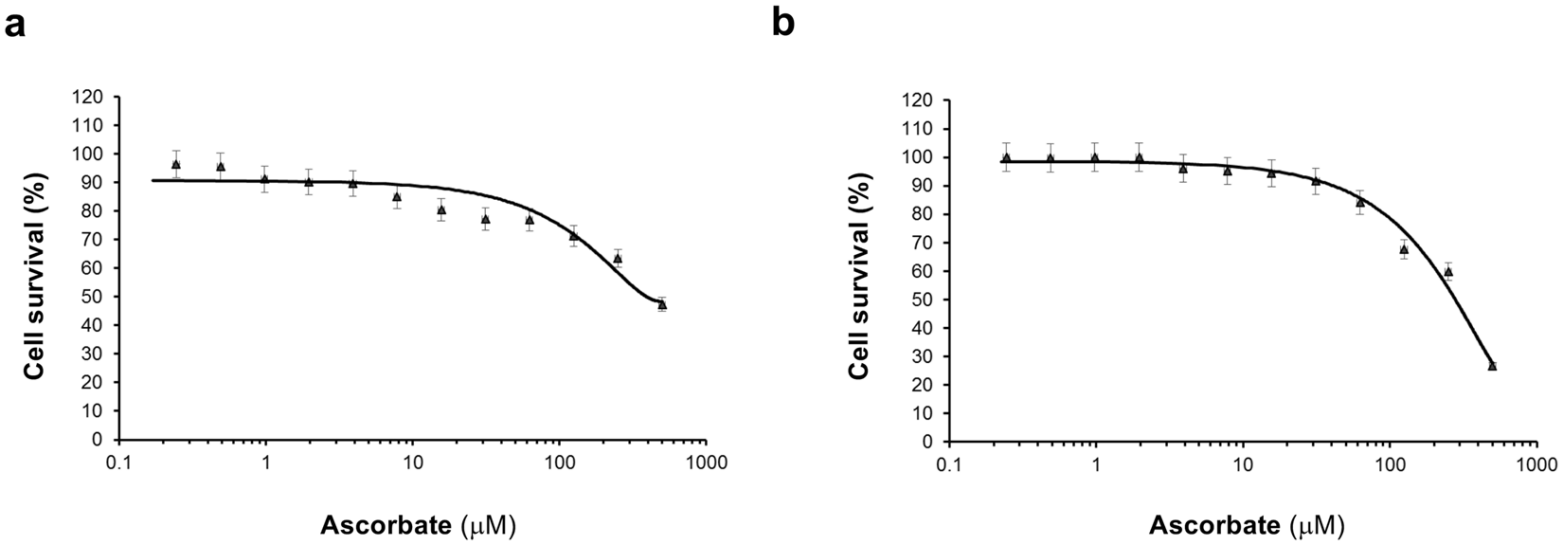
Melanocyte and melanoma cells show sensitivity to pharmacological dosage of ascorbate. Cell survival of healthy human melanocytes (HEMa) and metastatic melanoma cells (A2058) was measured after treatment with ascorbate over a wide range of concentrations for 72 hours. Data is presented as percent cell survival vs. ascorbate dosage. (**a**) Dose response for HEMa cells with a calculated EC50 of ~327 µM. (**b**) Dose response of metastatic melanoma cells (A2058) with a calculated EC50 of ~290 µM.

We Previously showed that ascorbate at a physiological concentration (100 μM) could largely restore 5 hmC content in A2058 melanoma cells, which reached to ~75% of the 5 hmC level observed in healthy melanocytes^19^. A pharmacological level (500 μM) of ascorbate did not exert additional benefits in 5 hmC restoration. However, we were puzzled that the partial restoration of 5 hmC had no obvious effect on cell proliferation. In this study, we first re-examined the survival of A2058 cells under treatment of different concentrations of ascorbate using an alternate cell viability assay. The result confirmed that ascorbate at 100 μM indeed did not affect the viability of A2058 cells. To our surprise, the EC50 of ascorbate in killing A2058 cells and normal melanocytes are very similar (Fig. 1, EC50 = 290 μM for A2058 cells and EC50 = 327 μM for normal melanocytes cells). This indicates that it might not be practical to apply high concentrations of ascorbate by intravenous injections in patients for cancer treatment as suggested before, because healthy melanocytes would be damaged as well.

**Figure 2:**
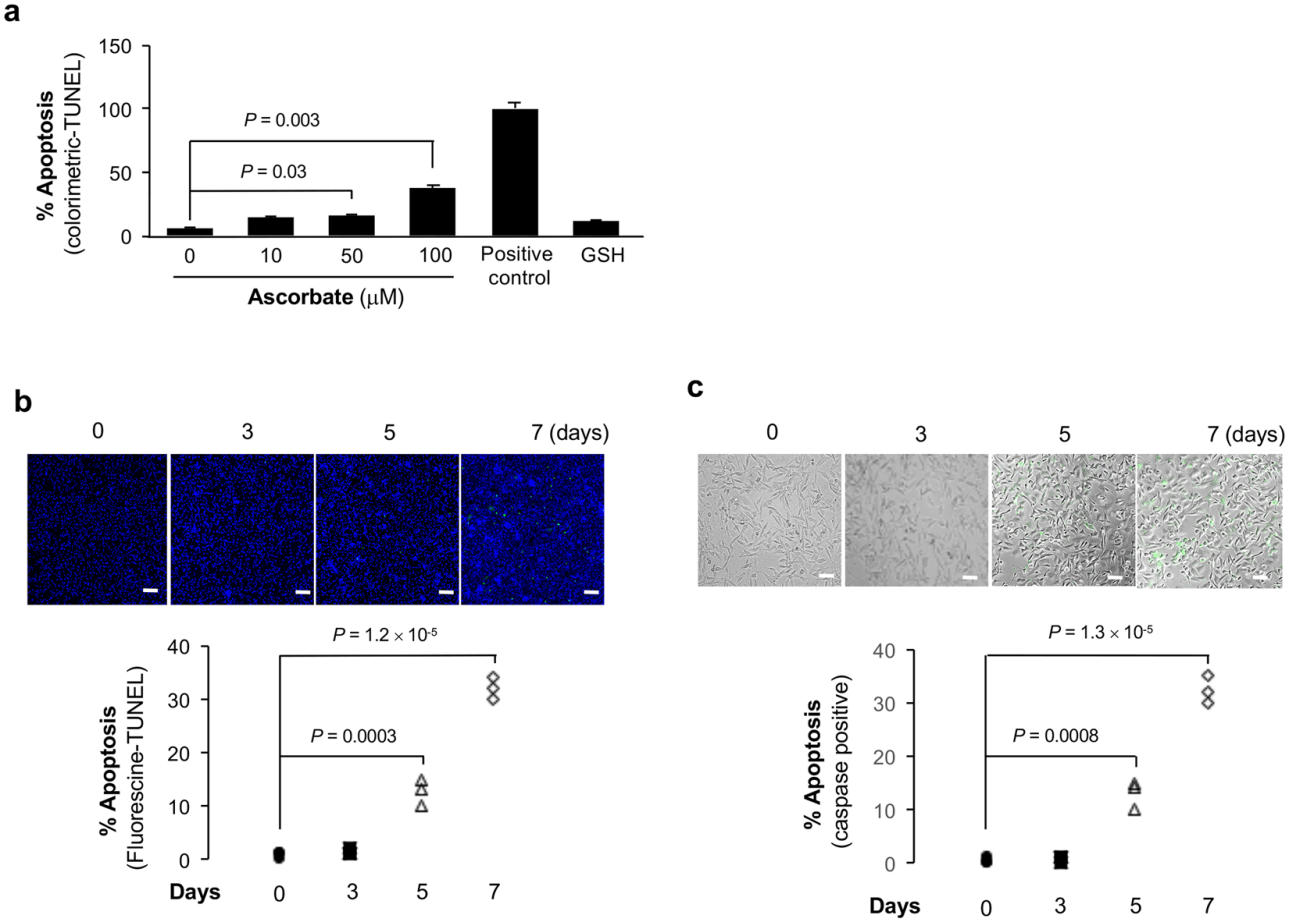
Treatment with ascorbate causes apoptosis in A2058 cells. (**a**) About 40% of the total cell population turned apoptotic after treatment with 100 µM ascorbate, but not 100 µM GSH, as measured by colorimetric TUNEL assay. (**b**) A2058 cells were treated with 100 µM of ascorbate for 7 days followed by Fluorometric TUNEL assay which stained apoptotic cells with Fluorescein. Images captured by fluorescent microscope show an increase in apoptotic cells in a time-dependent manner (bar = 100 µm). (**c**) A2058 cells treated with 100 µM ascorbate were stained with caspase 3/7 kit after 3, 5 and 7 days. Microscope images suggest an increase in caspase active cells with length of treatment (bar = 100 µm).

We then investigated if 5 hmC restoration by ascorbate consequently influences apoptosis of A2058 melanoma cells. We first used a colorimetric TUNEL assay to identify cells undergoing apoptosis. With close scrutiny, we observed ~40% of A2058 cells turned apoptotic after ascorbate (100 μM) treatment for 7 days, while ascorbate at 50 μM only induced less than 20% apoptotic cells (Fig. 2a). However, when A2058 cells were treated with glutathione (GSH), a potent antioxidant, there was no significant presence of apoptotic cells compared to the control group (ascorbate 0 μM; Fig. 2a). This suggests that the induction of apoptosis by ascorbate appears independent of its property as a general antioxidant. To validate these initial findings, a fluorometric TUNEL assay was conducted to visualize apoptotic cells after ascorbate (100 μM) treatment of A2058 cells for various periods of time. Fluorescein stained apoptotic cells were identified under microscope after 5 and 7 days, but not 3 days, of ascorbate (100 μM) treatment. A steady increase in apoptotic cells were captured with a turnout of ~40% after 7 days (Fig. 2b). To further confirm the induction of apoptosis by ascorbate, A2058 cells were probed for active caspase using caspase 3/7 staining. Corroborating aforementioned observations, caspase 3/7 stained cells were found after 5 and 7 days, but not 3 days, of ascorbate (100 μM) treatment (Fig. 2c). These results suggest that treatment of melanoma cells with ascorbate at a physiological concentration induces apoptosis, which also depends on caspase activation.

### Ascorbate Suppresses Clusterin Expression in Melanoma Cells

**Figure 3:**
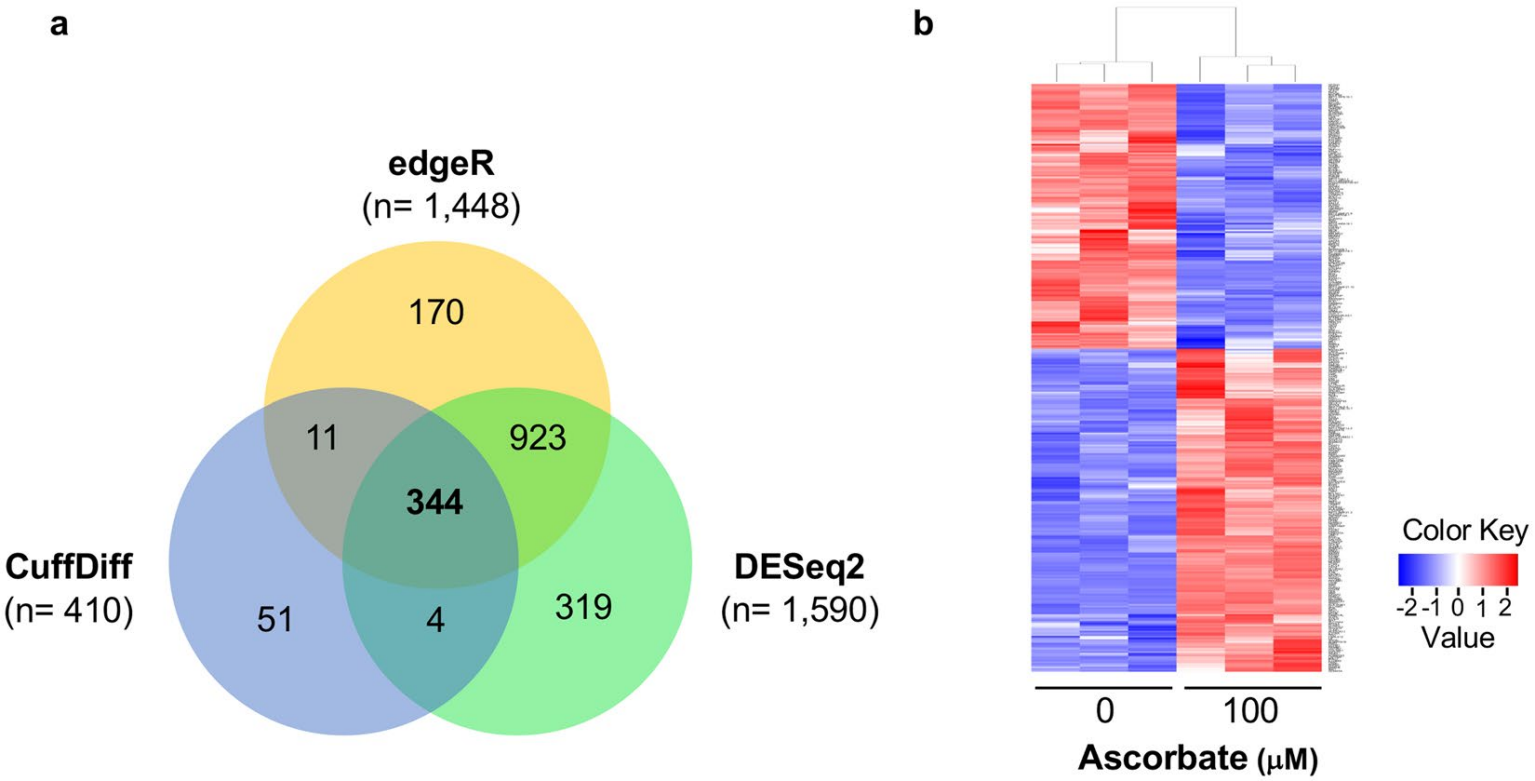
Ascorbate treatment shifts the transcriptome of A2058 cells. A2058 cells were treated with (100 µM) or without ascorbate for 7 days. RNA was extracted from both treated and untreated cells and subjected to high-throughput sequencing. (**a**) Venn diagram showing the number of significant genes as called by three different statistical algorithms: DESeq2, edgeR and CuffDiff. (**b**) Heatmap showing differentially expressed genes in A2058 cells. Colors represent Z-scores where downregulated transcripts are represented as blue and upregulated transcripts are represented as red.

We previously examined the transcriptome changes in A2058 cells after ascorbate (100 μM) treatment. However, none of the top genes (based on fold changes and *P* values) were directly involved in the apoptosis pathway^19^. To elucidate potential mechanism of ascorbate in inducing apoptosis in A2058 cells, we re-analyzed our existing RNA-seq using updated algorithms. DEseq2 was applied instead of the original version of DEseq. CuffDiff replaces Bayseq, which is often in dramatic disagreement with the other methods as shown in our previous results^19^. The updated analysis revealed that 1,590 genes were differentially expressed by DEseq2, 1,448 genes by edgeR, and 410 genes by CuffDiff respectively. A total of 344 genes including 20 non-coding RNAs (ncRNA) were significantly and differentially expressed between the ascorbate treated (100 μM) and untreated samples, as detected by all three algorithms (Fig. 3a). A clear shift in the transcriptome was observed after ascorbate treatment as shown in the heatmap (Fig. 3b). Of the 344 differential genes, 189 genes were upregulated while 155 genes were downregulated in A2058 cells by ascorbate treatment.

One of the most downregulated genes is Clusterin (CLU), which has long been implicated in protecting cells from apoptosis^22^. RNA-seq results showed that CLU transcripts decreased ~50% after ascorbate treatment.

**Figure 4:**
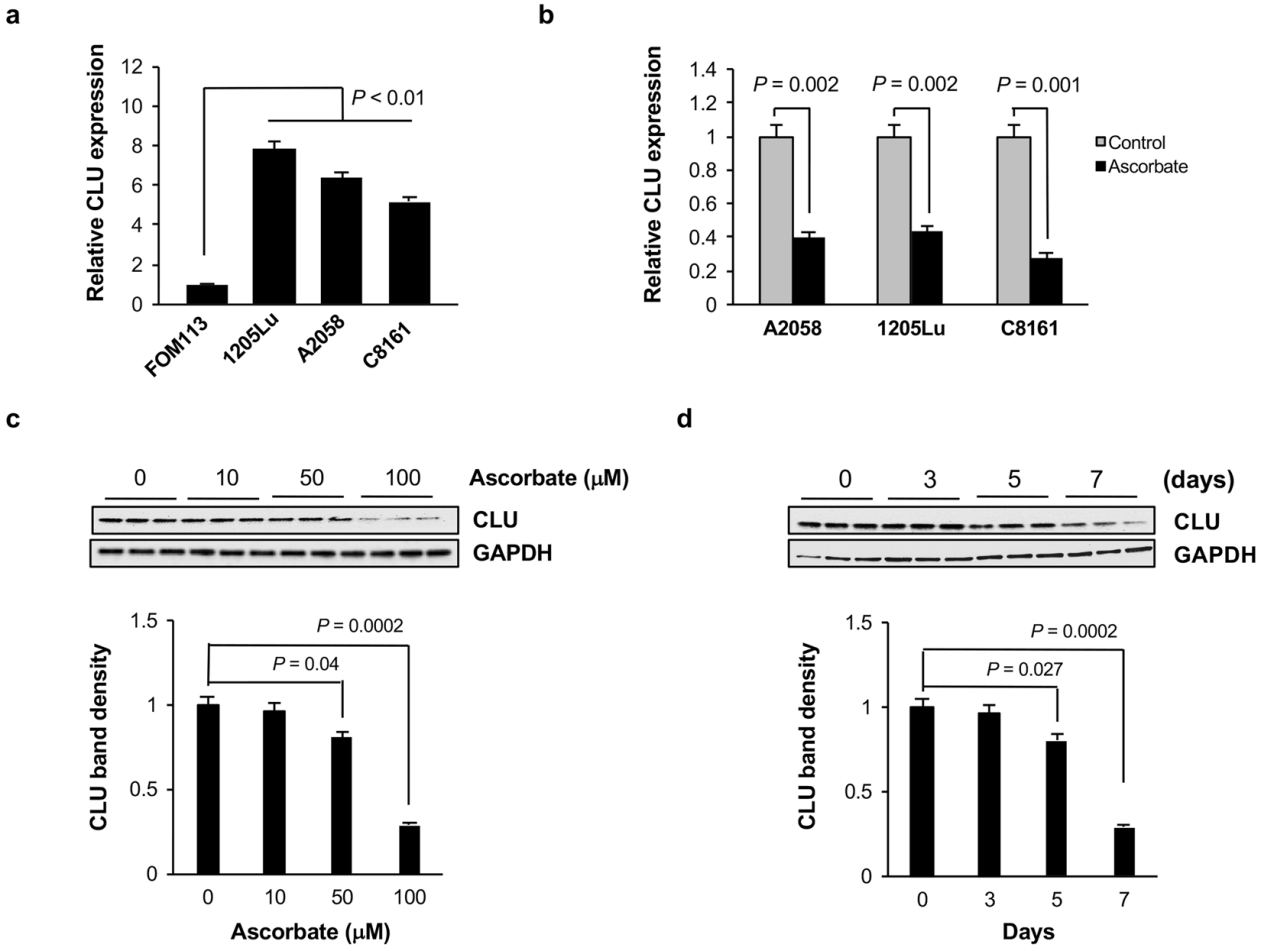
Clusterin expression is suppressed by ascorbate treatment (**a**) CLU transcripts were evaluated in healthy melanocytes (FOM-113) and metastatic melanoma cells (A2058, 1205Lu, C8161) by qRT-PCR. The level of CLU transcripts is higher in melanoma cells than healthy human melanocytes. (**b**) Ascorbate (100 µM) treatment decreases the level of CLU transcripts in melanoma cells (A2058, C8161 and 1205Lu), as shown by qRT-PCR. (**c**,**d**) Immunoblot results showing CLU expression at protein level in ascorbate treated A2058 cells. The expression of CLU is significantly lower when cells are treated with 100 µM of ascorbate. However, the effect of ascorbate treatment shows highest effect beyond 5 days of treatment.

We first examined CLU expression in healthy melanocyte and three melanoma cell lines available in our lab including A2058, 1205Lu, and C8161. Consistent with previous reports^22–26^, all three melanoma cell lines, derived from metastatic stages, showed significantly higher level of CLU mRNA (>5 fold) than in FOM-113, healthy melanocytes (Fig. 4a). We then applied quantitative RT-PCR (qRT-PCR) to verify the results of RNA-seq in A2058 cells. The results showed that ascorbate (100 µM) indeed reduced CLU mRNA level to ~40% of the controls (Fig. 4b). The suppression of ascorbate on CLU transcription was further validated in 1205Lu and C8161 cells.

To examine whether ascorbate treatment suppresses CLU at the protein level in A2058 cells, western blot analysis was implemented. With treatment of increasing doses of ascorbate (0 − 100 µM) for 7 days, the CLU protein level gradually decreased. No significant change was observed in CLU specific bands on western blot membrane after treatment with 10 µM ascorbate. Ascorbate at 50 µM reduced CLU protein to near 80% of the controls but ascorbate at 100 µM exerted a dramatic reduction in CLU protein, which was only at ~25% of the controls (Fig. 4c, S1). The effect of ascorbate on CLU protein expression also appeared in a time-dependent manner. After about 3 days of treatment with 100 μM ascorbate, we observed ~20% reduction in CLU protein, which eventually decreased as much as ~80% after 7 days of treatment (Fig. 4d, S2). Overall, these observations strongly indicate that ascorbate suppresses CLU expression in a dose-dependent and time-dependent manner.

### Ascorbate Reduces Cytoplasmic CLU While Largely Maintaining Nuclear CLU

**Figure 5:**
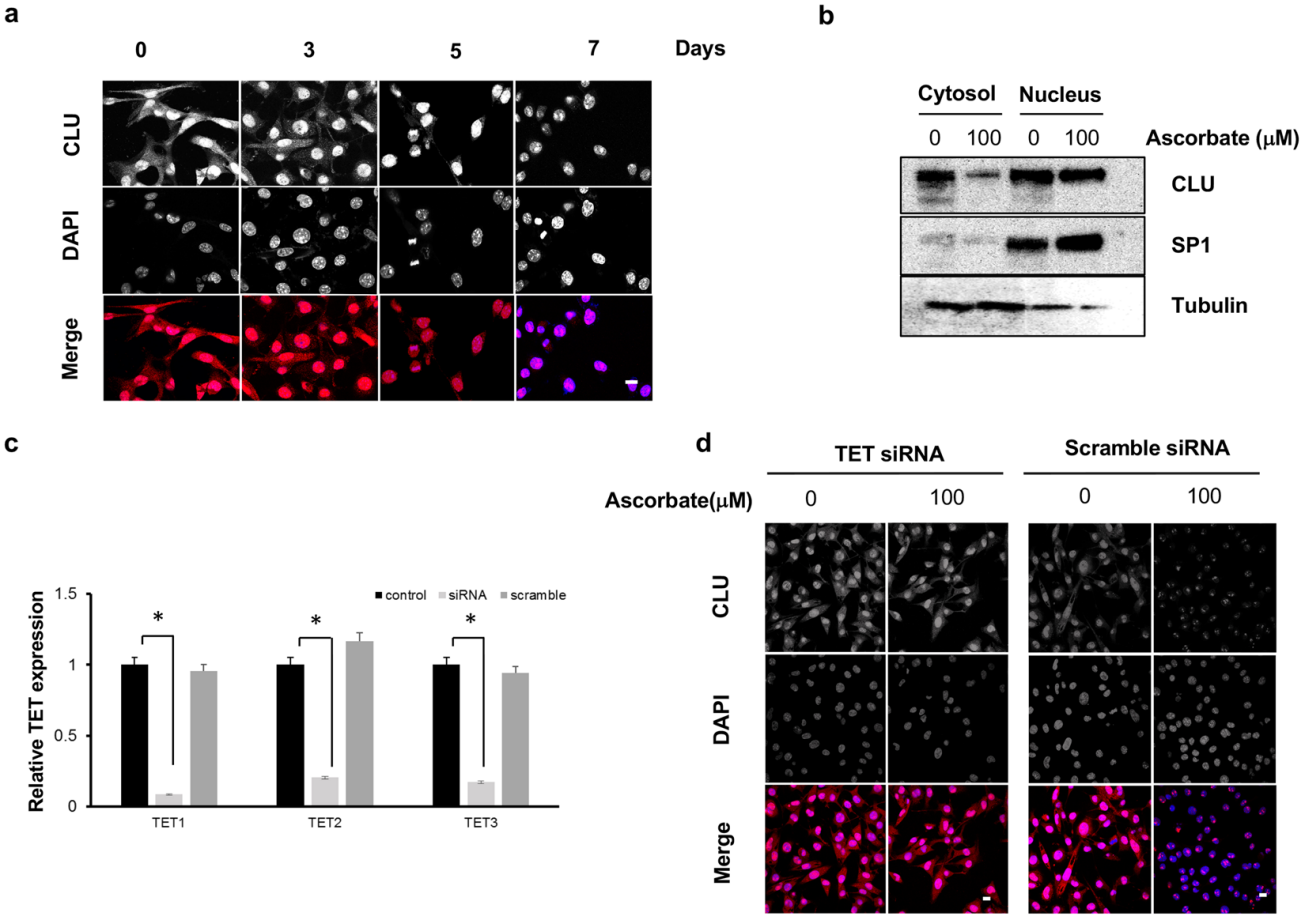
Cytoplasmic, but not nuclear, CLU is dramatically downregulated by ascorbate. (**a**) Immunofluorescent staining of A2058 melanoma cells treated with 100 µM ascorbate for 0, 3, 5 and 7 days shows a significant decrease in cytoplasmic but not nuclear CLU. (**b**) Immunoblot showing CLU in cytoplasmic and nuclear fractions of A2058 cells, treated with (100 µM) or without ascorbate. A downregulation in CLU from cytosol was evident after treatment with ascorbate. SP1 was probed as a nuclear extraction control and Tubulin as a cytoplasmic extraction control. (**c**) Relative fold change in TET1, TET2 and TET3 in un-transfected A2058 cells, pooled TET1/2/3 siRNA transfected A2058 cells and scramble transfected A2058 cells showing TET siRNA successfully inhibiting all three TET mRNA expression in A2058 cells. (**d**) CLU expression primarily localized in nucleus when treated with ascorbate (scramble RNAi) however both cytoplasmic and nuclear CLU existed in TET siRNA transfected A2058 cells with and without ascorbate treatment.

After verifying the suppressing effect of ascorbate on CLU at mRNA and protein levels, we examined subcellular localization of CLU protein due to the fact that CLU expression appears as different forms in different compartments^27^. Immunofluorescence results showed that CLU protein was distributed across cytoplasm and nucleus in the untreated A2058 cell population. After treatment with ascorbate at 100 µM for 3, 5 and 7 days, CLU protein gradually disappeared from the cytoplasm, whereas CLU was still observed in the nucleus though with a slightly tapered immunofluorescence signal (Fig. 5a).

Next, we evaluated CLU protein from the extractions of cytoplasmic and nuclear fractions of A2058 cells treated with (100 µM) or without ascorbate for 7 days. The success of cellular fractionation was confirmed by specific markers for nuclear (SP1) and cytoplasmic (Tubulin) proteins. Corroborating the immunofluorescence results, a steady decrease in cytoplasmic CLU was observed after ascorbate treatment. In contrast, ascorbate treatment only caused a slight reduction in nuclear CLU in A2058 cells (Fig. 5b, S3). Results of immunofluorescent staining and Western blot strongly suggest that ascorbate treatment reduces cytoplasmic CLU while nuclear CLU is largely maintained in melanoma cells.

To further evaluate whether modulation of CLU by ascorbate treatment is a direct impact of ascorbate in TETs-mediated DNA demethylation pathway, we utilized siRNA to knockdown the expression of TETs (TET1, TET2 and TET3) in A2058 cells. Compared to the scramble siRNA transfected controls, the mRNA level of all three TETs were dramatically reduced by siRNA as shown by qRT-PCR (Fig. 5c). CLU expression in cytoplasm remained largely unchanged after ascorbate (100 µM) treatment for 5 days in TETs knockdown cells. Whereas control A2058 cells (untransfected or transfected with scramble siRNA) showed a significant reduction in cytoplasmic CLU after the same ascorbate treatment (Fig. 5d). These results indicate that the modulation of CLU expression by ascorbate is likely through the TETs-mediated DNA demethylation pathway.

### Ascorbate treatment induces Bax activation

**Figure 6:**
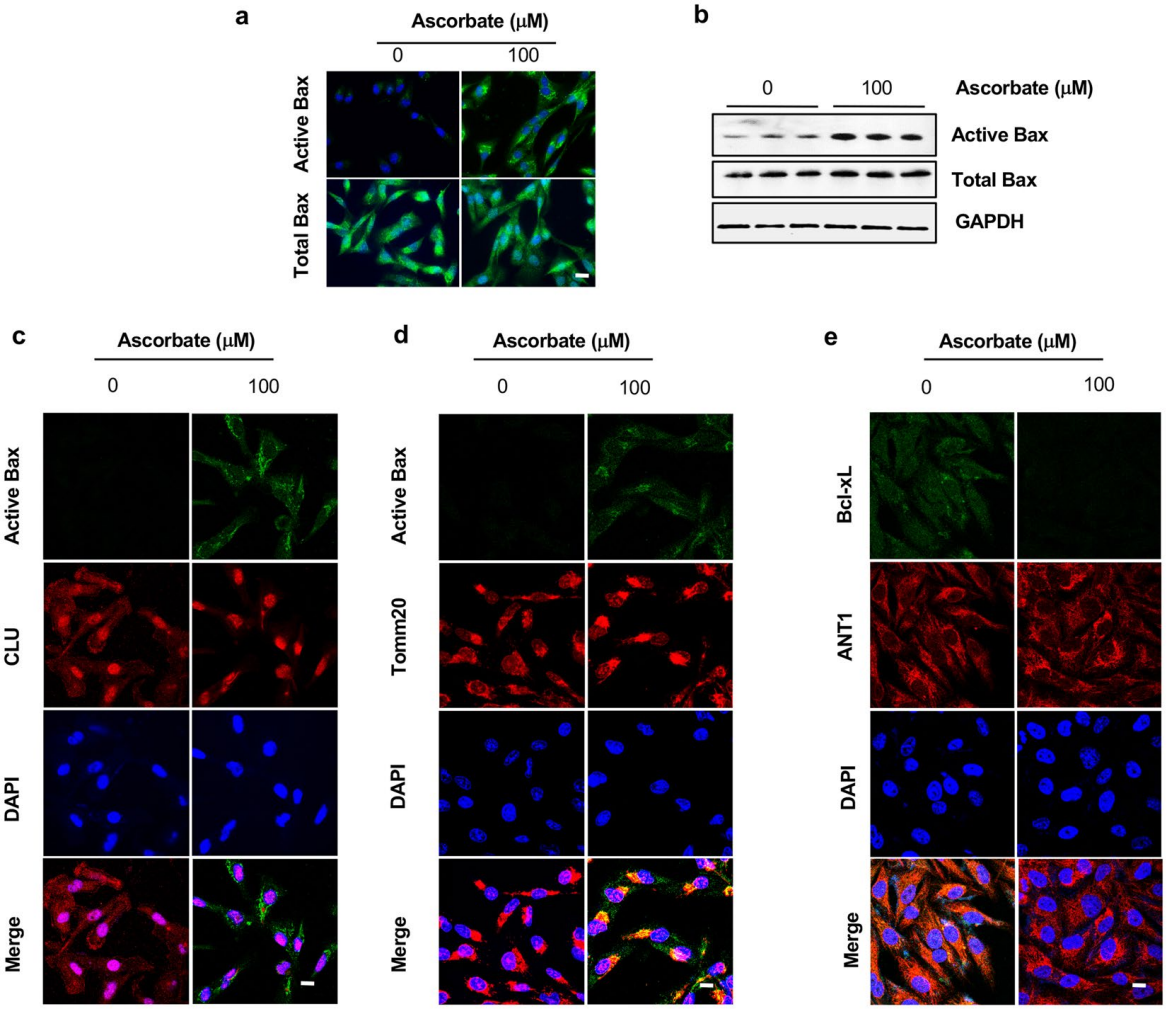
Bax activation and mitochondrial translocation in A2058 cells increases after treatment with ascorbate. (**a**) Immunofluorescence shows that ascorbate treatment induces active Bax protein in A2058 cells whereas total Bax remains unaltered. (**b**) Immunoblot showing active Bax increases after ascorbate treatment in A2058 cells as captured by Active Bax antibody (6A7) mediated immuno-precipitation. (**c**) Immunofluorescent staining showing that in presence of higher expression of CLU, active Bax could not be detected in A2058 cells. However, after ascorbate treatment, active Bax increases and CLU decreases from A2058 cells. (**d**) Ascorbate treatment induces active Bax co-localization with mitochondrial protein Tomm20 in A2058 cells. (**e**) Ascorbate treatment diminished Bcl-xL colocalization with mitochondrial protein ANT1 in A2058 cells.

Studies have showed that CLU in the cytoplasm stabilizes Bax protein in the process of forming a protein complex with another protein Ku70^28^, thus rendering the Bax protein inactive and inhibiting Bax-mediated apoptosis^28^. Since our observations so far indicated that cytoplasmic CLU is decreased in ascorbate-treated A2058 cell, we therefore examined the status of Bax activation. By immunofluorescent detection, there was no obvious active Bax in untreated cells. However, after treatment with 100 µM ascorbate for 7 days, A2058 cells displayed strong signal of the activated Bax (Fig. 6a). To further confirm the observation from immunofluorescent staining, we enriched active Bax using immunoprecipitation with an antibody against a specific BAX epitope, an active form at 6A7. The immunoprecipitation showed an obvious increase in active Bax bands after ascorbate treatment (Fig. 6b, S4). Results from these two experiments demonstrated a significant increase in active Bax after ascorbate treatment.

Next, we sought to determine a potential correlation of downregulated cytoplasmic CLU with Bax activation in response to ascorbate treatment in A2058 cells. Confocal microscopy images revealed that ascorbate (100 µM) treatment caused appearance of active Bax and disappearance of cytoplasmic CLU in A2058 cells at the same time (Fig. 6c). After treatment with ascorbate, CLU protein was mainly localized in the nucleus. There was no obvious co-localization of active Bax and CLU in the cytoplasm, suggesting that they were inversely regulated.

**Figure 7:**
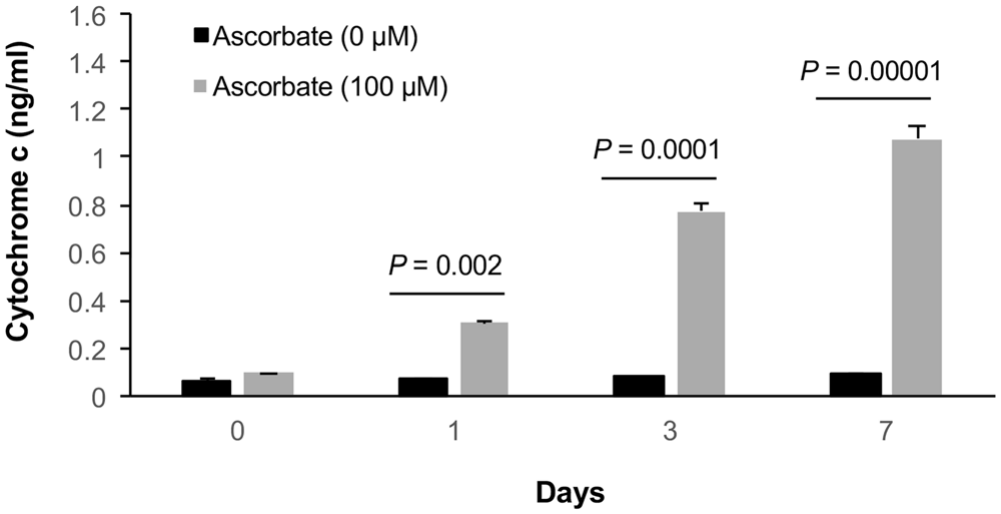
Ascorbate enhances cytochrome c release from A2058 cells. A time course of ascorbate treatment is followed by quantitative analysis of cytochrome c release from A2058 cells using ELISA. Cytochrome c release is increased by prolonged ascorbate treatment from ~0.3 ng/mL after one day of treatment to ~1 ng/mL after 7 days of treatment.

It is known that the active form of Bax protein localizes on the mitochondrial membrane. In subsequent experiments we looked into the localization of active Bax induced by ascorbate. Indeed, the active Bax co-localized with a mitochondrial outer membrane marker Tom20 post treatment, suggesting that active Bax is localized on the mitochondrial membrane when cells are treated with ascorbate (Fig. 6d). Accompanying the activation of Bax, nuclear CLU sequesters the anti-apoptotic protein Bcl-xL and promotes apoptosis by activation of caspase-3 and cytochrome c release^29^. In untreated A2058 cells, Bcl-xL protein at a much higher level was colocalized with inner mitochondrial protein ANT1. The mitochondrial Bcl-xL was subsequently diminished by ascorbate treatment (Fig. 6e). We then analyzed and quantified the released Cytochrome c from A2058 cells treated with ascorbate (100 μM) for different periods of time. Cytochrome c release was indeed increased significantly in response to ascorbate treatment from day 3 onwards (Fig. 7). These results suggest that ascorbate enhances apoptosis in melanoma cells by decreasing the cytoplasmic CLU and maintaining the nuclear CLU, which sequesters Bcl-xL, activates Bax and caspases, and releases cytochrome c.

## Discussion

Clusterin, as a multifunctional chaperone, is expressed in various tissues. There are two major transcript isoforms due to alternative splicing, which consequently yield two protein isoforms with distinct functions. The secreted form of the CLU protein appears in cytoplasm functions as an extracellular chaperone, which prevents protein aggregation. Upon cellular stress, secreted CLU can also translocate from the secretory compartments to the cytosol. Zhang *et al*. found that intracellular secreted CLU inhibited apoptosis by interfering with BAX activation in the mitochondria^27^. In contrast, the nuclear form of the CLU protein promotes apoptosis principally by sequestering Bcl-xL and releasing Bax, which accompanies caspase-3 activation and cytochrome c release^29^. Thus, CLU protein exerts opposite effects on cell apoptosis depending on its cellular localizations, i.e. anti-apoptotic secreted CLU and pro-apoptotic nuclear CLU^27^.

Overexpression of CLU has been identified in different types of cancer such as lung, prostate, and gastric cancers, which correlates with advanced tumor stage, poor prognosis, and resistance to chemotherapies^30^. CLU is expressed in primary and metastatic melanoma cases^23–25^, while the high expression of CLU is identified in invasive desmoplastic melanoma^26^. Furthermore, studies confirmed that CLU staining is mainly cytoplasmic and only occasionally in the nucleus in melanoma as shown by immunohistochemistry^25, 26^. These suggest that elevated CLU, especially secreted CLU, disrupts the balance of apoptosis regulation and leads to malignant transformation of melanocytes and melanoma progression.

Much effort has been focused on finding a means to inhibit CLU because overexpression of CLU appears to be involved in drug resistance^31^. Antisense oligonucleotides, by targeting CLU, can decrease CLU levels and sensitize melanoma cells to standard chemotherapeutic drugs^32^. Some of those antisense oligonucleotides are under testing in phase III clinical trials.

We have demonstrated here the ability of ascorbate to effectively suppress CLU transcription and decrease protein level in melanoma cells. Specifically, ascorbate treatment dramatically decreases cytoplasmic CLU but largely maintain the level of nuclear CLU. Thus, ascorbate induces apoptosis in melanoma cells by inhibiting the anti-apoptotic function of secreted CLU as well as retaining pro-apoptotic function of nuclear CLU. Previous studies showed the apoptosis induction in murine and human melanoma cells at high pharmacological concentrations by elevating intracellular reactive oxygen species generation (ROS) levels^33, 34^. However, it could be extremely difficult to reach high ascorbate concentration (up to 10 ~ 16 mM) *in vivo* even by intravenous injection. Thus, their relevance to clinical care of melanoma patients might be compromised. In contrast, this research focuses on ascorbate at 100 µM, a concentration which can be conveniently reached by diet and dietary supplement. Furthermore, the mechanism of apoptosis induction by ascorbate at high pharmacological concentrations or a physiological concentration could be different. Rather than elevating ROS to damage cells, ascorbate at 100 µM changes gene expression, such as CLU, by acting as a cofactor for TETs to enhance DNA demethylation.

The initial hint of ascorbate mediated suppression of CLU expression came from direct investigation of apoptosis related genes in RNA-seq analysis of A2058 cells treated with ascorbate. Consistent with previous studies, the level of CLU mRNA is much higher in melanoma cell lines than in healthy melanocytes^23–26^. The suppression of ascorbate on CLU transcripts was then verified in different melanoma cell lines, and confirmed at the protein level (Fig. 4). Interestingly, after ascorbate treatment, CLU protein in the cytoplasm and nucleus was not equally decreased. We observed that secreted CLU in cytoplasm almost disappeared while nuclear CLU still existed in the nucleus though not as strong as in the control cells (Fig. 5). We also confirmed the essential role of TET enzymes in modulating CLU expression upon ascorbate treatment (Fig. 5). Corroborating with these observations, we found active Bax localized in the mitochondrial membrane after ascorbate treatment where as anti-apoptotic Bcl-xL is sequestered from the mitochondrial membrane at the same time (Fig. 6). These separate lines of evidence support the notion that ascorbate induces apoptosis in melanoma cells by suppressing secreted CLU, which further activates Bax and Caspases.

The suppression of CLU expression could be mediated by the partial restoration of 5 hmC content in melanoma cells after ascorbate treatment, as shown in our previous study^19^. It is known the 5 hmC exerts bi-directional effects on transcription^35^, which is also exemplified in our RNA-seq data with 189 genes upregulated and 155 genes downregulated (Fig. 3). Besides TETs-mediated DNA demethylation, ascorbate could also regulate histone demethylation by serving as a cofactor for the JmjC domain-containing histone demethylases, which, like TETs, also belong to the iron and 2-oxoglutarate -dependent dioxygenase family^36–38^. Both DNA demethylation and histone demethylation are involved in regulating CLU transcription^39, 40^, and potentially alternative splicing, which could be instrumental to explain the differential effect of ascorbate on secreted CLU and nuclear CLU. Thus, elucidating the detailed epigenetic, or other, mechanism of ascorbate in suppressing CLU expression, especially the secreted CLU isoform, is ensured in future studies.

There is a long controversial history of ascorbate as a treatment for cancer, possibly due to the fact that it is nearly impossible to quantitatively control dietary ascorbate consumption in human subjects. In contrast, animal studies clearly show the benefit of ascorbate supplementation in treating melanoma^41, 42^.

Furthermore, conventional studies apply mega doses of ascorbate in order to produce free radicals or other byproducts that hopefully can “kill” cancer^43^. Here, we show that high concentrations of ascorbate could be toxic to non-cancerous cells such as healthy melanocytes. Therefore, the benefit of intravenous injections of mega doses of ascorbate may be limited. In our research, ascorbate is used to compensate for the downregulated SVCT2 in order to enhance and possibly maximize the catalytic activity of TETs in melanoma cells, thus rebuilding an epigenetic profile towards that of healthy melanocytes. By altering gene expression, this epigenetic action of ascorbate helps decrease melanoma malignancy, exemplified here by CLU downregulation.

In conclusion, ascorbate induces apoptosis in melanoma cells by suppressing CLU expression. This study highlights a possible use of ascorbate in preventing melanoma progression and a co-treatment of advanced melanoma by sensitizing melanoma cells to standard chemotherapies.

## Methods

### Cell Culture and Treatment

Human melanocyte line FOM-113, derived from a healthy human subject, and melanoma cell lines derived from metastatic phase (C8161, 1205Lu) were gifts from Dr. M. Herlyn (The Wistar Institute). Metastatic melanoma cell line A2058 was purchased from ATCC (Manassas, VA). Human Epidermal melanocytes, adult (HEMa) were purchased from Thermo Scientific (Weltham, MA). Melanoma cells were maintained under a 5% CO_2_ atmosphere in RPMI medium (Life technologies, Carlsbad, CA) supplemented with 10% heat inactivated fetal bovine serum (FBS), 100 Units/ml penicillin and 100 µg/ml of streptomycin. Melanocytes were cultured in 254 medium supplemented with Human melanocyte growth supplements (Thermo Scientific, Waltham, MA). During treatments, cells were seeded for 24 h, and subsequently treated with L-Ascorbic acid (Sigma-Aldrich, St. Louis, MO). Culture media were replaced by fresh media with or without ascorbate every 24 hours to avoid accumulation of unabsorbed ascorbate.

### Cell Survival assay

A2058 and HEMa cells were maintained in culture flasks at 37 °C with 5% CO_2_. The cells were seeded in white bottom 384-well plates (Thermo-scientific) at a density of 5 × 10^2^ cells per well in 25 μL medium and were allowed to attach and grow for 24 hours. Afterwards, a 5 μL solution of different concentrations of ascorbate were added to each well in order to generate dose response curves from a 10-point 1:3 dilution series starting at a nominal test concentration of 0.1 μM. The ascorbate concentration at each treatment point was repeated in triplicate. The cells were incubated for 72 hours and then live cell counts were measured by CellTiter-GLo assay (Promega, Madison, WI) following manufacturer’s protocol. The Envision Multi-label Reader (Perkin Elmer, Waltham, MA) was used to measure the luminescence produced by the live cells. For each cell line, percent cell survival was plotted against ascorbate concentration. The reported IC50 values were generated from fitted curves by solving for the X-intercept value at the 50% inhibition level of the Y-intercept value.

### Apoptosis Assays

A2058 melanoma cells were seeded in 24 well plates with coverslips and treated with L-ascorbic acid (Sigma-Aldrich St. Louis MO) at different concentrations for 7 days. Glutathione (GSH) was also used to treat cells in a control experiment at a concentration similar to the highest concentration of ascorbate. Apoptotic cells were detected at the end of the treatment utilizing the following three different techniques: (1) colorimetric TUNEL was measured by an *in situ* apoptosis detection kit (Trevigen, Gaithersburg, MD); (2) fluorescein-based TUNEL was visualized by a cell death detection kit (Sigma-aldrich, St. Louis, MO); and (3) caspase activation was evaluated by incubating cells with CellEvent Caspase-3/7 Green Detection Reagent for 30 min prior to imaging. Each well of TUNEL positive cells or caspase active cells was imaged using a 2D fluorescent microscope system and analyzed with Image J. All experiments were repeated at least 3 times.

### Quantification of Cytochrome C

A2058 cells were treated with Ascorbate (100 μM) for 3, 5 or 7 days. Equal number of cells (1.5 × 10^6^) were collected from each treatment point and Cytochrome c was measured using Cytochrome c Human ELISA kit (abcam, Cambridge, UK). For control (0 days), cells were collected immediately after adding ascorbate.

### RNA-seq Reanalysis

Total RNA was extracted from A2058 cells using the RNeasy Mini Kit (Qiagen, Valencia, CA). A Bioanalyzer 2000 was used to measure the quality of RNA. All samples’ RNA integrity numbers (RIN) were above 9. Whole transcriptome sequencing was carried out at the Sequencing Core of John P. Hussman Institute of Human Genomics at the University of Miami using the Epicentre Ribo-Zero Human/Mouse/Rat kit (Epicentre, Madison, WI). Briefly, after ribosome RNA (rRNA) was depleted, sequencing libraries were constructed following the standard Illumina protocols and were subsequently processed by a Hiseq2000 sequencing system (125 bp paired-end reads, 4 samples per lane; Illumina, San Diego, CA). Raw read data was first run through quality control metrics using FastQC (http://www.bioinformatics.babraham.ac.uk/projects/fastqc/). Sequence reads were aligned to the human transcriptome (GRCh38, Ensembl.org), realigned around known splice junctions in the transcriptome and quantified using the STAR aligner^44^. Statistical significances were determined using 3 different differential expression calculators: edgeR, DESeq2, and CuffDiff^45–47^. To reduce false positives, only genes that gave an adjusted P –value below 0.05 across all three differential expression calculators were considered “differential”.

### RNAi sequences and transfection

RNA interference (RNAi) sequences directed against human TET1 (5′-CUUUAAUGGCUGUAAGUUU-3′), human TET2 (5′-GCCUUGAGCAGUAAUAUU-3′), human TET3 (5′-AGGCCAAGCUCUACGGGAA-3′), nontarget scramble (5′-GCCUUGAGCAGUAAUAUUU-3′), were designed and synthesized by Dharmacon (Lafayettte, CO). Prior to RNAi transfection, A2058 cells were plated in growth medium without antibiotics at 30 to 50% confluence. Transfection of RNAi sequences (10 nM concentration for each TETs, 30 nM total final concentration) was performed using Lipofectamine 2000 (Invitrogen, Carlsbad, CA), as specified by Invitrogen. Cells were maintained for 5 days (transfected on Day 0 and Day 3). Media was changed 6 hours after transfection to eliminate the toxic effects of transfecting reagents.

### Quantitative RT-PCR

The relative expression of Clusterin mRNA in melanoma or melanocyte cells before and after ascorbate treatment (100 µM for 7 days) was determined by real-time quantitative RT-PCR. Total RNA was extracted from all replicates of treated and untreated cells using QIAGEN RNeasy mini kit (QIAGEN, Valencia, CA) and the yield was quantified using a Nanodrop 8000 spectrophotometer. cDNA was synthesized from 1 µg of total RNA with the Super Script III First-Strand Synthesis System (Invitrogen, Carlsbad, CA). Real-time Quantitative PCR assay was performed in with 100 ng of diluted cDNA, perfecta Syber green super mix (Quanta Biosciences Gaithersburg, MD) and qPCR primers. The sequences of primers used are Clusterin (Forward: 5′-GAGCTCCAGGAAATGTCCAATCAGG-3′; Reverse: 5′-CCTCTCATTTAGGGCATCCTCTTTCTTC-3′), TET1 (Forward: 5′-AATGGAAGCACTGTGGTTTG-3′; Reverse: 5′-ACATGGAGCTGCTCATCTTG-3′), TET2 (Forward: 5′-AATGGCAGCACATTGGTATG-3′; Reverse: 5′-AGCTTCCACACTCCCAAACT-3′) TET3 (Forward: 5′-GAGGAGCGGTATGGAGAGAA-3′; Reverse: 5′-AGTAGCTTCTCCTCCAGCGT-3′) and GAPDH (Forward: 5′-TGGACCTGACCTGCCGTCTA-3′; Reverse: 5′-CCCTGTTGCTGTAGCCAAATTC-3′ internal control) primers on an Applied Biosystems 7900HT. Relative gene expression was determined using the 2^−ΔΔ*C*^_T_ method^48^. Mean *C*_T_ of triplicate measures were computed for each sample. Sample mean *C*_T_ of GAPDH was subtracted from the sample mean *C*_T_ of Clusterin (Δ*C*_T_). The Δ*C*_T_ of the sample with no treatment was selected as a calibrator and subtracted from the mean Δ*C*_T_ of each experimental sample (ΔΔ*C*_T_). 2^−ΔΔ*C*^_T_ yields fold change in gene expression of the gene of interest normalized to the internal control and relative to the calibrator sample. Statistical significance of differences in Clusterin expression levels between cells treated with or without ascorbate were assessed by Student *t* test, at α = 0.05.

### Cell Lysis, Subcellular fractionation and Immunoblotting

For immunoblot analysis cells were washed twice with PBS and then lysed with RIPA cell lysis buffer (50 mM Tris-HCl, 150 mM NaCl, 0.1% SDS, 0.5% sodium deoxycholate, 1% NP40) in the presence of protease and phosphatase inhibitors. For Subcellular fractionation, 2 × 10^8^ cells were collected for the extraction and NE-PER nuclear and cytoplasmic extraction kit (Thermo-Scientific, Waltham, MA) was used. Lysates were collected with cell scrapers and cleared by centrifugation. Prior to SDS-PAGE, cell lysates were resuspended in SDS sample buffer (60 mM Tris–HCl, 1% SDS, 10% glycerol, 0.05% bromophenol blue, pH 6.8, with 2% β-mercaptoethanol). Samples were subjected to 10% SDS-PAGE (Bio-Rad, Hercules, CA) and transferred to PVDF membranes (Bio-Rad) for immunoblotting. Transfer efficiency was determined by Ponceau S staining (Sigma-Aldrich, St. Louis, MO). PVDF membranes were incubated with blocking solution (TBS containing 0.1% Tween 20 and 5% BSA) and were probed with gene specific antibodies (Clusterin; H-330, GAPDH; 0411, Santa Cruz, Dallas, Tx). Protein bands were detected using chemiluminescence kit (Millipore, Billerica, MA). ImageJ (NIH) was used to quantify immunoblot results. Digital images were captured and loaded to ImageJ, then Clusterin specific bands as well as GAPDH bands were assessed in each immunoblot membrane. The ratio of Clusterin signal to GAPDH was calculated for treatment point which served as an index of Clusterin expression.

### Co-Immunoprecipitation

For active Bax direct immuno precipitation assay, Pierce direct IP kit (Thermo-Scientific, Waltham, MA) was used following manufacture’s protocol. Briefly, 6A7 Bax monoclonal antibody (Santa Cruz, Dallas, TX) was immobilized on an aldehyde-activated beaded agarose resin. The antibody resin was then incubated with treated and untreated A2058 cell lysate in order to pull down active Bax. Next, active Bax protein was eluted and samples were run on immunoblot. Further, on immunoblot membrane, the immunoprecipitated Bax was detected using Bax (2D2) (Santa Cruz, Dallas, TX) antibody. Total Bax and GAPDH were also probed from the treated and untreated A2058 sample fractions.

### Immunofluorescence

A2058 cells were seeded in 6-well culture dish with coverslips for 24 hours before treatment. After completion of treatment, coverslips with cells were washed three times with cold PBS. The cells were fixed for 10 minutes at room temperature with 4% paraformaldehyde in PBS, and permeabilized for 5 minutes with 0.2% Triton X-100 PBS. Cells were then treated with 1 N HCL at 37 °C for 15 min to permeabilize the nucleus followed by neutralization with 100 mM of Tris-HCL followed by 5% BSA. The cells were then incubated with the primary antibodies (CLU and Bax as mentioned before, TOMM20: EPR15581 (abcam, Cambridge, UK); Bcl-xL: 2762 (Cell signaling, Danvers, MA); ANT1: ab110322 (abcam, Cambridge, UK) at 1:50 dilution in PBS over night at 4 °C, followed by the secondary antibodies (for CLU:Alexa fluor 568 goat anti-rabbit, for Bax, Alexa fluor 488 donkey anti-mouse; for BcL-xL, Alexafluor 488 goat anti-rabbit; for Tomm20, Alexa fluor 568 goat anti-rabbit, for ANT1, Alexa fluor 555 donkey anti-mouse) at 1:250 dilution in PBS for another hour. Each step was preceded by three washes in PBS. To stain the nucleus, cells were incubated with 40 µg/ml 4′,6-diamidino-2-phenylindole (DAPI) for 20 minutes at room temperature. The coverslips were then mounted on glass slides and examined at room temperature with a Zeiss LSM 710 confocal laser scanning microscope. Images were processed with the help of Merge-color application of NIH ImageJ software.

### Statistical Analysis

All observations in this study were analyzed in triplicate and each experiment was repeated three times. Values represent the mean ± SEM of three independent experiments. To compare two groups, student’s *t* test was used and *P* < 0.05 was considered as statistically significant.

## Electronic supplementary material

Supplemental information
