# Supplementary material for: Ascorbate induces apoptosis in melanoma cells by suppressing Clusterin expression
Source: Sci Rep. 2017 Jun 16;7:3671. doi: 10.1038/s41598-017-03893-5 (PMC5473908; doi:10.1038/s41598-017-03893-5)
Supplement: Supplementary file 1 — Supplemental information [file 41598_2017_3893_MOESM1_ESM.pdf]

Supplementary Information

Ascorbate induces apoptosis in melanoma cells by suppressing Clusterin expression

Sushmita Mustafi[1], David W. Sant[1], Zhao-Jun Liu[2,3], Gaofeng Wang[1,3,4§]

[1]John P. Hussman Institute for Human Genomics, Dr. John T. Macdonald Foundation Department of Human Genetics; [2] Department of Surgery; [3] Sylvester Comprehensive Cancer Center; [4] Dr. Nasser Ibrahim Al-Rashid Orbital Vision Research Center, University of Miami Miller School of Medicine, Miami, FL 33136, USA.

[§] Correspondence should be addressed to:

Gaofeng Wang, Ph.D.

John P. Hussman Institute for Human Genomics

University of Miami Miller School of Medicine

1501 NW 10th Ave, Biomedical Research Building 608

Miami, FL 33136, United States of America

E-mail: gwang@med.miami.edu

Telephone: (305) 243-7318

Fax: (305) 243-2703

Supplementary Figure 1

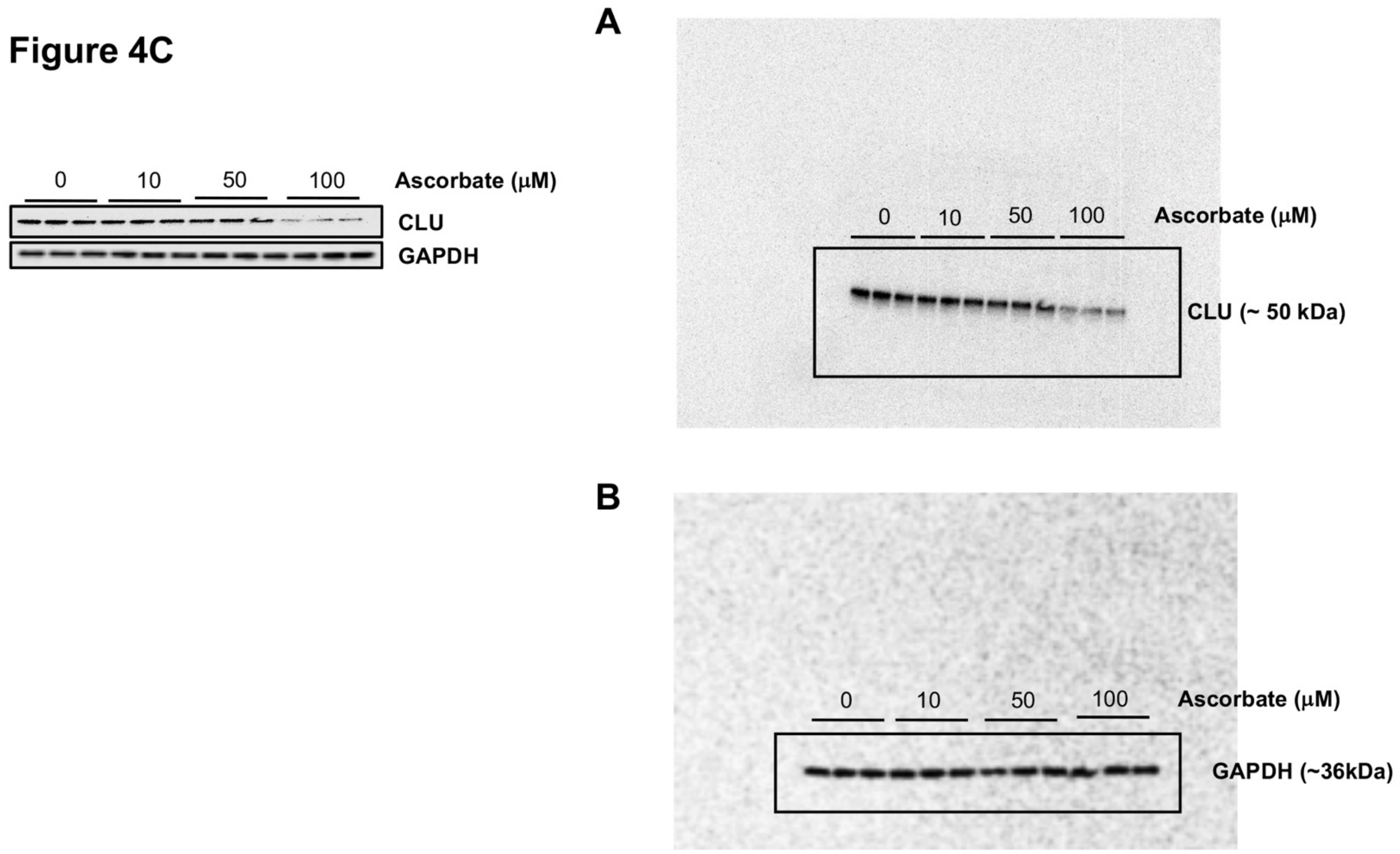

Supplementary Figure 1. Immunoblot membrane images of CLU expression in A2058 cells treated with different concentrations of ascorbate (0, 10, 50 and 100 μM). The cropped images are shown in **Figure 4C**. **(A)** Immunoblot membrane was probed with anti-CLU primary antibody and was imaged in FluorChemE system through Chemiluminescence channel. **(B)** Immunoblot membrane was then stripped and re-probed with anti-GAPDH primary antibody, subsequently imaged in FluorChemE system through Chemiluminescence channel.

Supplementary Figure 2

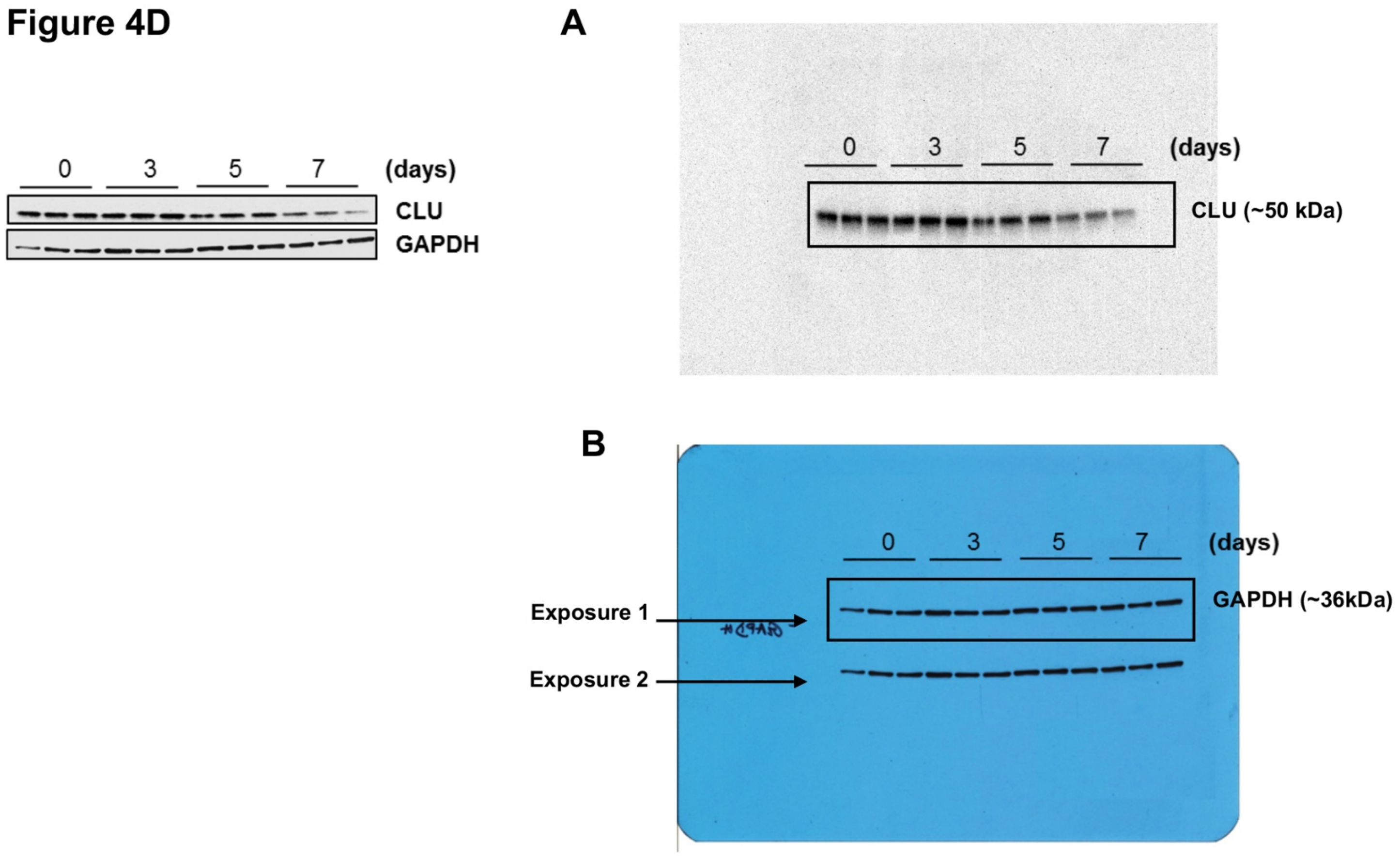

Supplementary Figure 2. Immunoblot membrane images of CLU expression in A2058 cells treated with ascorbate (100 µM) for a period of 0, 3, 5 and 7 days. The cropped images are shown in **Figure 4D**. **(A)** Immunoblot membrane was cut between ladder 50kDA and 36 kDA. The top half was probed with anti-CLU primary antibody and was imaged in FluorChemE system through Chemiluminescence channel. **(B)** The bottom half of the Immunoblot membrane probed with anti-GAPDH primary antibody was imaged on X-ray film for immunoblot imaging and developed with two different exposures.

Supplementary Figure 3

Figure 5B

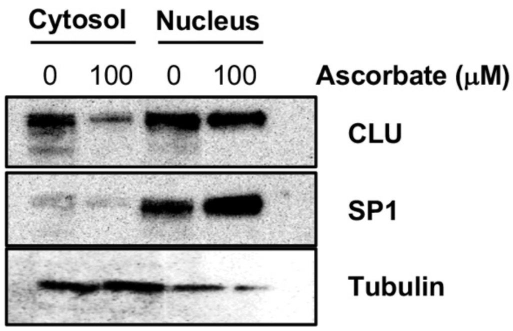

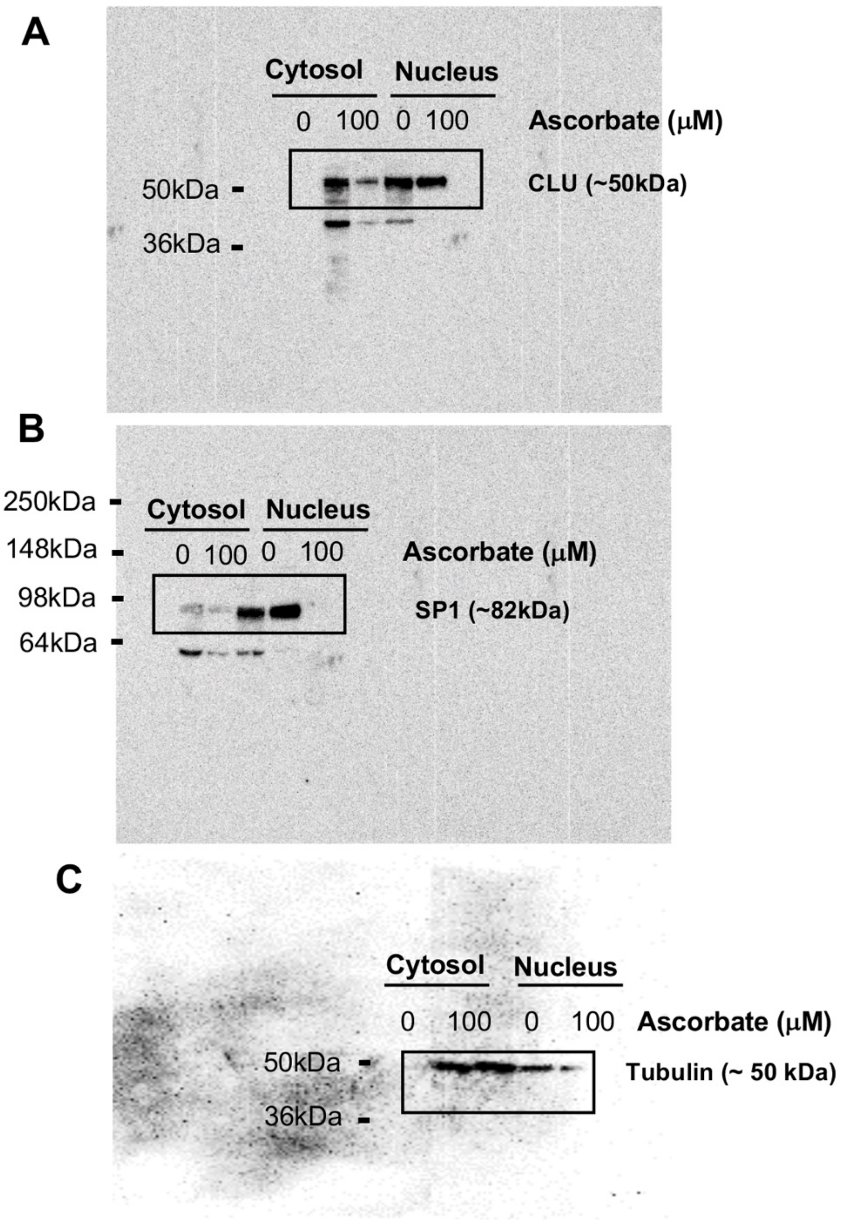

Supplementary Figure 3. Images of immunoblot showing CLU distribution in nuclear and cytoplasmic fractions with or without ascorbate treatment. The cropped images are shown in **Figure 5B**. **(A)** Immunoblot membrane was cut near 64kDa ladder size. The bottom part was probed with anti-CLU primary antibody and was imaged in FluorChemE system through Chemiluminescence channel. **(B)** Top part of the immunoblot membrane was probed with anti-SP1 primary antibody and imaged in FluorChemE system through Chemiluminescence channel. **(C)** Bottom part of the immunoblot membrane was further re-probed with anti-tubulin primary antibody and was imaged in FluorChemE system through Chemiluminescence channel.

Supplementary Figure 4

Figure 6B

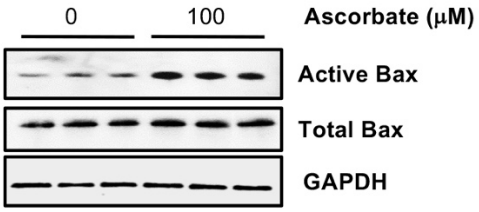

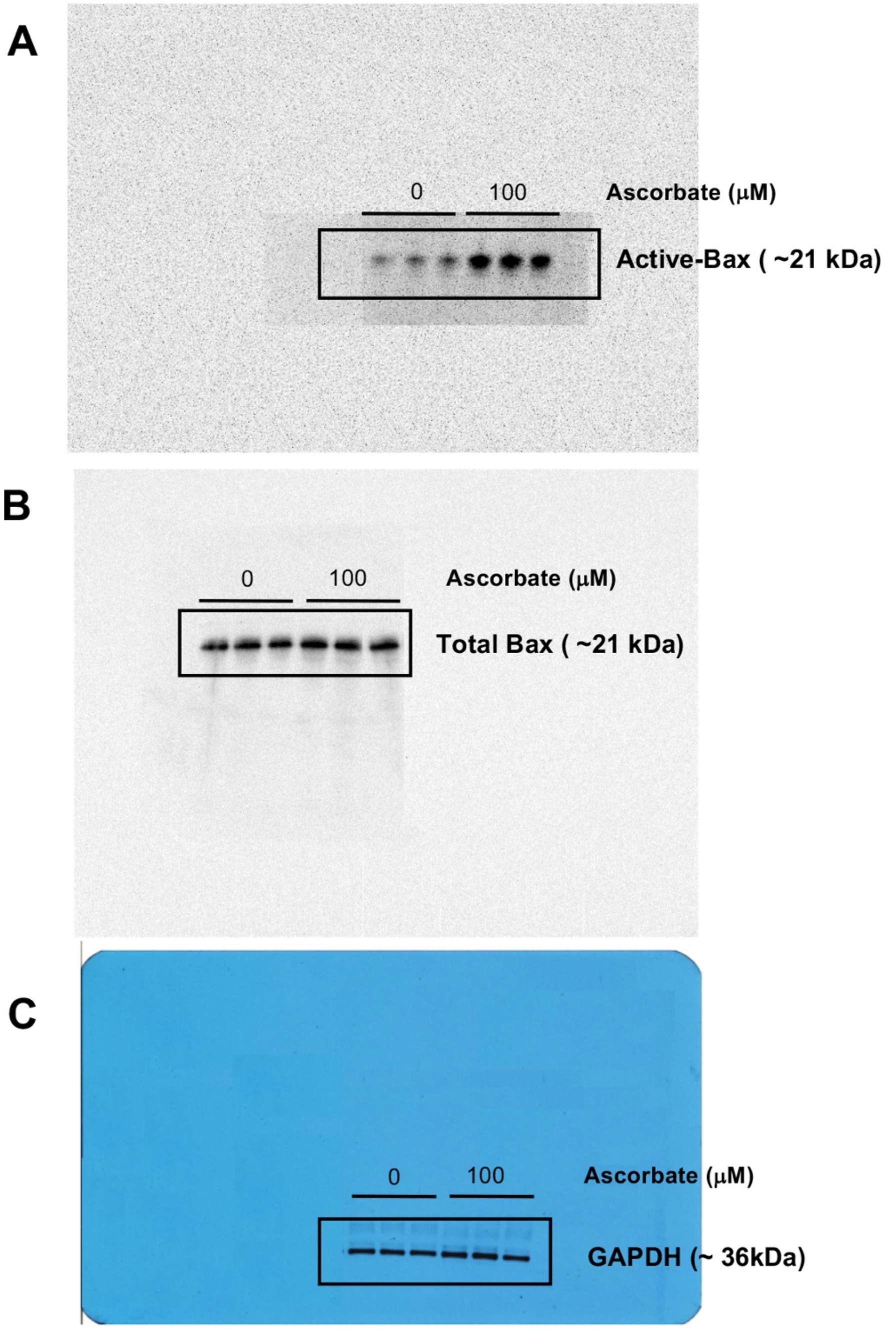

Supplementary Figure 4. Images of Immunoblot results showing active Bax and total Bax in A2058 cells with or without ascorbate treatment (100 μM). The cropped images are shown in **Figure 6B**. **(A)** Immunoblot membrane was cut into two parts along a line just above 36kDa ladder. The bottom half was probed with anti-active Bax (active monomer 6A7) primary antibody and was imaged in FluorChemE system through Chemiluminescence channel. The top half was probed for CLU proteins (data not shown). **(B)** Immunoblot membrane from another run of SDS-PAGE gel with same sample loading order was probed with anti-total Bax primary antibody and was imaged in FluorChemE system through Chemiluminescence channel. **(C)** Immunoblot membrane from active Bax probe was stripped and re-probed with anti-GAPDH primary antibody was imaged on X-ray film for Immunoblot imaging and developed in medical film developer.
